# Electrochemical evaluation of anti-CRP/CRP interaction for the molecular diagnosis of the cardiovascular risk

**DOI:** 10.1186/1753-6561-8-S4-P37

**Published:** 2014-10-01

**Authors:** Vinícius Rodovalho, Anderson Lemos, João Marcos Madurro, Ana Graci Brito-Madurro

**Affiliations:** 1 Laboratory of Biomaterials, Institute of Genetics and Biochemistry, Federal University of Uberlândia, Uberlândia, Brazil; 2 Laboratory of Polymeric Films and Nanotechnology, Institute of Chemistry, Federal University of Uberlândia, Uberlândia, Brazil

## Background

The C-reactive protein (CRP or PTX1) is an acute phase protein, expressed by hepatocytes, that is regarded as a clinical marker for infection and has been increasingly used as a risk indicator in chronic inflammatory diseases [1]. Atherosclerosis is an inflammatory disease that remains a major cause of morbidity and mortality. Its progression is associated with the accumulation of lipoproteins in the endothelium, establishing plaques whose rupture may result in the formation of a thrombus, arising several cardiovascular complications [2]. Studies show there is a correlation between the risk of cardiac events (such as myocardial infarction) and increasing levels of CRP [3]. Currently, there are several methods to detect CRP, including immunoassays and agglutination. However, these methods are not sensitive enough, time-consuming or cost-ineffective [4]. Therefore, new diagnosis methods are being developed. Electrochemical biosensors are small devices that combine the selectivity of biochemical molecular recognition with the sensitivity of electrode transducers, being remarkable for their specificity, speed, portability and low cost [5]. In this work, a graphite electrode surface is modified with poly(3-aminotiophenol) and a specific antibody (anti-CRP), aiming the detection of its antigen (CRP), through electrochemical methods.

## Methods

The monomer solution (3-aminotiophenol) was prepared in 0.50 mol.L^-1 ^sulfuric acid solution for electropolymerization by potential cycling between -0.4 V and +1.0 V vs. Ag/AgCl at 50 mV.s^-1^. This solution was prepared freshly, before each electropolymerization and deaerated with ultra pure nitrogen before use for 40 minutes. After electropolymerization, the modified ectrode was rinsed in deionized water to remove unreacted monomer. The electrochemical polymerization was performed through cyclic voltammetry in a three-compartment cell using a potentiostat from CH Instruments model 420A. The solution containing the antibody probe (anti-CRP, 50µg.mL^-1^) was added onto the surface of the modified electrode. Then, the modified electrode containg poly(3-aminotiophenol):anti-CRP was incubated in solution of glycine to block and prevent non-specific adsorption. Next, a solution containing the antigen target (CRP, 150 µg.mL^-1^) was applied onto the produced biomaterial. The indicator of the interaction anti-CRP:CRP, 4-aminoantipyrine (4-AAP), was added to the system and its oxidation peak was monitored. After each step, the electrodes were rinsed with 0.1 mmol.L^-1 ^phosphate buffer pH 7.4 to eliminate not-bound biomolecules. These detections were carried out in a one-compartment electrochemical cell, through differential pulse voltammetry.

## Results and conclusions

The cyclic voltammogram of polymerization showed an increase in current values with increasing number of potential scans, reflecting the coverage of the electrode surface by the polymeric film. The 4-AAP oxidation peak was detected in the potential of +0,3V. The current values of oxidation peaks for the antibody probe (anti-CRP):antigen target (CRP) system were higher when compared to the system containing the antibody in the absence of the antigen. This result indicate that the immunosensor poly(3-ATP):anti-CRP produced discriminate the presence of the CRP target. Therefore, the anti-CRP:CRP interaction onto the modified electrode surface is a promising platform for the molecular diagnostic of the inflammatory process and cardiac diseases.
